# Midbrain–hindbrain malformations in patients with malformations of cortical development and epilepsy: A series of 220 patients

**DOI:** 10.1016/j.eplepsyres.2013.05.001

**Published:** 2013-09

**Authors:** Giorgi Kuchukhidze, Florian Koppelstaetter, Iris Unterberger, Judith Dobesberger, Gerald Walser, Julia Höfler, Laura Zamarian, Edda Haberlandt, Kevin Rostasy, Martin Ortler, Thomas Czech, Martha Feucht, Gerhard Bauer, Margarete Delazer, Stephan Felber, Eugen Trinka

**Affiliations:** a Department of Neurology, Medical University of Innsbruck, Anichstrasse 35, Innsbruck 6020, Austria; b Department of Neurology, Paracelsus Medical University of Salzburg, Christian Doppler Klinik, Ignaz Harrer Strasse 79, Salzburg 5060, Austria; c Department of Radiology, Medical University of Innsbruck, Anichstrasse 35, Innsbruck 6020, Austria; d Department of Pediatrics IV, Division of Neuropediatrics, Medical University of Innsbruck, Anichstrasse 35, Innsbruck 6020, Austria; e Department of Neurosurgery, Medical University of Innsbruck, Anichstrasse 35, Innsbruck 6020, Austria; f Department of Neurosurgery, Medical University of Vienna, Währinger Gürtel 18-20, Vienna 1090, Austria; g Department of Pediatrics, Medical University of Vienna, Währinger Gürtel 18-20, Vienna 1090, Austria; h Institute of Radiology and Nuclear Medicine, Stiftungsklinikum Mittelrhein, Johannes Müller-Strasse 7, Koblenz 56068, Germany

**Keywords:** Epilepsy, Cortical dysplasia, MRI, Developmental disorders, Midbrain–hindbrain

## Abstract

Midbrain–hindbrain malformations (MHM) may coexist with malformations of cortical development (MCD). This study represents a first attempt to investigate the spectrum of MHM in a large series of patients with MCD and epilepsy. We aimed to explore specific associations between MCD and MHM and to compare two groups of patients: MCD with MHM (wMHM) and MCD without MHM (w/oMHM) with regard to clinical and imaging features.

Two hundred and twenty patients (116 women/104 men, median age 28 years, interquartile range 20–44 years at the time of assessment) with MCD and epilepsy were identified at the Departments of Neurology and Pediatrics, Innsbruck Medical University, Austria. All underwent high-resolution MRIs (1.5-T) between 01.01.2002 and 31.12.2011. Midbrain–hindbrain structures were visually assessed by three independent raters.

MHM were seen in 17% (38/220) of patients. The rate of patients wMHM and w/oMHM differed significantly (*p* = 0.004) in three categories of MCD (category I – to abnormal neuronal proliferation; category II – to abnormal neuronal migration; and category III – due to abnormal neuronal late migration/organization): MCD due to abnormal neuronal migration (31%) and organization (23%) were more commonly associated with MHM compared to those with MCD due to abnormal neuronal proliferation (9%). Extensive bilateral MCD were seen more often in patients wMHM compared to those w/oMHM (63% vs. 36%; *p* = 0.004). In wMHM group compared to w/oMHM group there were higher rates of callosal dysgenesis (26% vs. 4%; *p* < 0.001) and hippocampal abnormalities (52% vs. 27%; *p* < 0.001). Patients wMHM were younger (median 25 years vs. 30 years; *p* = 0.010) at the time of assessment and had seizure onset at an earlier age (median 5 years vs. 12 years; *p* = 0.043) compared to those w/oMHM. Patients wMHM had higher rates of learning disability (71% vs. 38%; *p* < 0.001), delayed developmental milestones (68% vs. 35%; *p* < 0.001) and neurological deficits (66% vs. 47%; *p* = 0.049) compared to those w/oMHM.

The groups (wMHM and w/oMHM) did not differ in their response to antiepileptic treatment, seizure outcome, seizure types, EEG abnormalities and rate of status epilepticus. Presence of MHM in patients with MCD and epilepsy is associated with severe morphological and clinical phenotypes.

## Highlights

• We assessed midbrain–hindbrain in a large series of cortical malformation patients.
• Midbrain–hindbrain malformations are commonly linked to cortical malformations.
• Midbrain–hindbrain malformations are associated with severe clinical phenotype.

## Introduction

Malformations of cortical development (MCD) represent a major cause of medically refractory epilepsy in children and adults (Barkovich et al., 2009, Fauser et al., 2006, Krsek et al., 2008, Leventer et al., 2010, Palmini et al., 1991). MCD are classified based upon the step at which neuronal development was first disrupted: (I) abnormal neuronal proliferation, (II) abnormal neuronal migration, (III) abnormal late migration/cortical organization (Barkovich et al., 2009). Increased or decreased proliferation of cortical neurons at early stages of brain development results in microcephaly, macrocephaly, tuberous sclerosis, focal cortical dysplasia (FCD) type II with balloon cells, hemimegalencephaly or dysplastic tumors (ganglioglioma, dysembryoplastic neuroepithelial tumor) – MCD of category I. MCD of category II – e.g. periventricular nodular heterotopia (PNH) or subcortical band heterotopia (SBH) are caused by disruption of neuronal migration from ventricular areas, where neurons proliferate, to cortical surface (Barkovich et al., 2009). Polymicrogyria (PMG), schizencephaly and FCD type I represent MCD of category III with aberrant late migration/cortical organization (lamination, gyration) (Barkovich et al., 2009).

MCD may be associated with other cerebral structural anomalies such as abnormalities of hippocampus (Kuchukhidze et al., 2010), corpus callosum (Hetts et al., 2006) or midbrain–hindbrain malformations (MHM) (Patel and Barkovich, 2002). These anomalies may determine clinical presentation of patients with MCD and therefore, play an important role in their management including epilepsy surgery. In a recent study we hypothesized, that the underlying pathophysiology of the MCD could be responsible for both MCD and associated hippocampal pathology (Kuchukhidze et al., 2010).

MHM represent a heterogeneous group of posterior fossa anomalies (Patel and Barkovich, 2002). Several attempts to classify MHM have been proposed based either on morphology, disrupted developmental stages or genetic background (Barkovich et al., 2009, D‘Arrigo et al., 2005, Demaerel, 2002, Niesen, 2002, Patel and Barkovich, 2002, Sztriha and Johansen, 2005, Utsunomiya et al., 2006).

The patterns of midbrain–hindbrain involvement in some MCD, like lissencephaly, have been well documented (Boycott et al., 2005, Hong et al., 2000, Kato et al., 1999). MHM may assist in classifying MCD and linking them with genetics and pathology (Jissendi-Tchofo et al., 2009, Pisano et al., 2012). Previous studies on series of patients with MHM analyzed the spectrum of MCD associated with cerebellar malformations (*n* = 70) (Patel and Barkovich, 2002), clinical features of epilepsy in patients with MHM (*n* = 10) (Recio et al., 2007) or disorders of cognitive and affective development in patients with MHM (*n* = 27) (Tavano et al., 2007). However, no studies have explored the types of MHM in a large and heterogeneous cohort of patients with MCD.

This study represents a first attempt to investigate the spectrum of MHM in a large series of patients with MCD and epilepsy. We also aimed to explore specific associations between MCD and MHM and to compare two groups of patients: MCD with MHM (wMHM) and MCD without MHM (w/oMHM) with regard to clinical and imaging features.

## Methods

Two hundred and twenty-eight patients with the MRI diagnosis of MCD were identified in the database of the Departments of Neurology and Pediatrics, Innsbruck Medical University, Austria. The database included over 7000 patients with epilepsy at the time of this analysis. All patients had epilepsy. They had at least two MRIs between 01.01.2002 and 31.12.2011. Three children underwent presurgical assessment and epilepsy surgery at the Departments of Pediatrics and Neurosurgery, Medical University of Vienna, Austria. Twelve patients were also assessed at the Department of Neurology, Paracelsus Medical University, Salzburg, Austria.

MCD were classified based on nomenclature proposed by Barkovich et al., 2005, Barkovich et al., 2012: category I – due to abnormal neuronal proliferation; category II – due to abnormal neuronal migration and category III – due to abnormal neuronal late migration/organization. This most widely used classification of MCD is primary based on imaging features of MCD. However, it increasingly incorporates genetic, molecular biological and embryological grounds of MCD reflecting its underlying neurobiology (Barkovich et al., 2005, Barkovich et al., 2012). Dysembryoplastic neuroepithelial tumor and ganglioglioma (total *n* = 33) were also included in the analysis under the category “developmental tumors” as they are incorporated in the classification of MCD (Barkovich et al., 2005, Barkovich et al., 2012).

### Imaging

All patients underwent high-resolution MRI (1.5-T) using a standard protocol which all patients with epilepsy undergo at our institution. MRI sequences included T1-weighted three-dimensional axial magnetization prepared rapid gradient echo (MPRAGE) images with and without intravenous contrast application, axial and coronal T2-weighted turbo spin echo, T1-weighted inversion recovery, T2-weighted fast fluid attenuated inversion recovery (FLAIR) and diffusion weighted sequences. Coronal T2-weighted and FLAIR slices were 3 mm thick and were acquired at 90° perpendicular to the long axis of hippocampus.

### Definition of terms, criteria for assignment to MHM and selection of a study population

The database of the Departments of Neurology and Pediatrics of the Innsbruck Medical University was screened for patients with MCD and epilepsy. All MRIs were analyzed retrospectively for detection and categorization of MHM by two independent raters: a neurologist (GK) experienced in neuroimaging of patients with MCD and a neuroradiologist (FK). Both raters were blinded for the clinical information (except for the knowledge that all patients had MCD and epilepsy).

**Figure 1:**
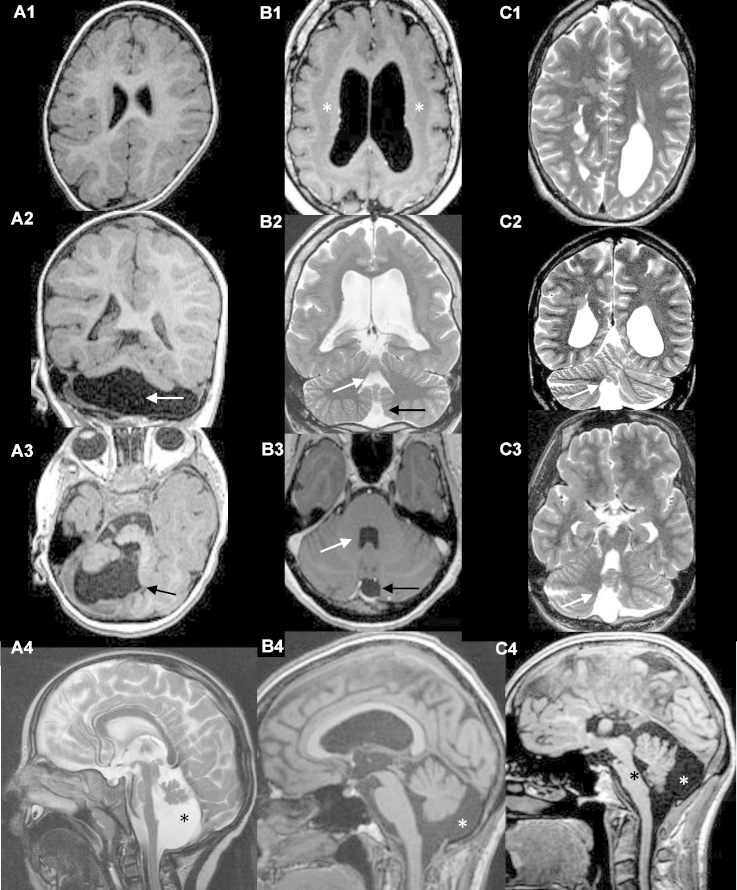
Cerebellar hypoplasia. First column (A1–4) – severe cerebellar hypoplasia. (A1) Axial T1-weighted image shows microcephaly, (A2) coronal T1 – weighted image shows enlarged posterior fossa (white arrow) with severely hypoplastic vermis and cerebellar hemispheres, (A3) T1-weighted axial image with large posterior fossa cerebro-spinal fluid (CSF) collection (black arrow), (A4) midline sagittal T2-weighted image shows enlarged IV ventricle, large collection of CSF in posterior fossa (asterisk) and hypoplastic vermis. Second column (B1–4) – isolated hypoplasia of cerebellar vermis. (B1) Asterisks show subcortical band heterotopia on T1-weighted axial image; coronal T2-weighted (B2) and axial T1-weighted (B3) images show enlarged IV ventricle (white arrow) and large collection of CSF in posterior fossa (black arrow), (B4) T1-weighted sagittal image with hypoplastic vermis and large collection of CSF in posterior fossa (white asterisk). Third column (C1–4) – severe hypoplasia of brainstem and cerebellum. (C1) Axial T2-weighted image, periventricular nodular heterotopia on the right; coronal (C2) and axial (C3) T2-weighted images show hypoplastic cerebellar vermis with enlarged IV ventricle and large collection of CSF in posterior fossa (white arrow), (C4) atrophic pons (black asterisk), hypoplastic vermis with enlarged collection of CSF in posterior fossa (white asterisk) and agenesis of corpus callosum on sagittal T1-weighted image.

**Figure 2:**
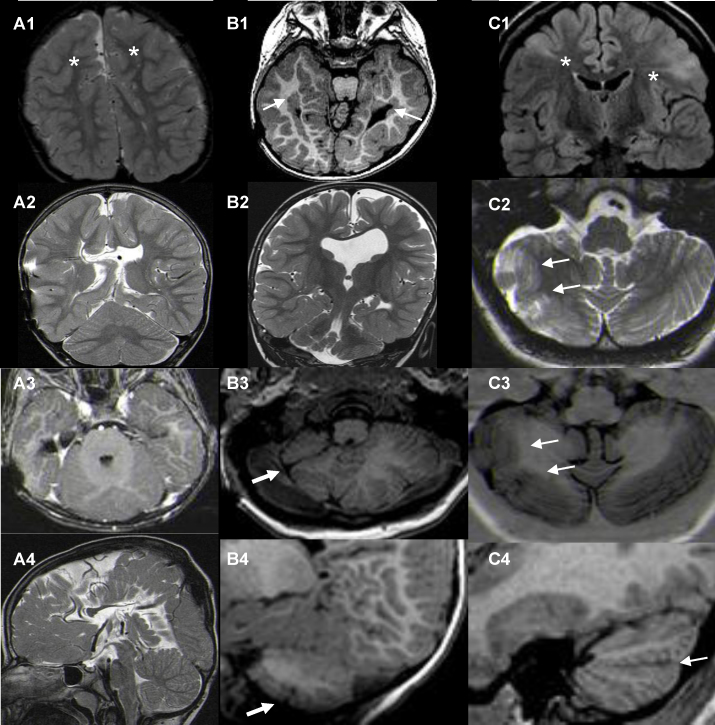
Cerebellar dysplasia. First column (A1–4) – rhombencephalosynapsis. (A1) Axial T2-weighted image with bilateral frontal polymicrogyria (asterisk); (A2) T2-weighted coronal and (A3) T1-weighted axial images show dorsally fused cerebellar hemispheres and superior cerebellar peduncles; (A4) T2-weighted sagittal image. Second column (B1–4) – cerebellar polymicrogyria with cleft. (B1) Axial T1-weighted image with bilateral periventricular nodular heterotopia along temporal horns of lateral ventricles (thin white arrows); (B2) coronal T2-weighted, (B3) axial T1-weighted and (B4) sagittal T1-weighted images show irregular structure of cerebellum in three different planes. (B2) Hypoplastic/partially infolded hippocampi. Cleft with overlying polymicrogyria (thick white arrows in B3 and B4) of the right cerebellar hemisphere (right hemisphere is smaller compared to the left one). Third column (C1–4) – cerebellar polymicrogyria with cleft. (C1) subcortical tubers on coronal FLAIR image (white asterisk); (C2) axial T2-weighted and (C3) T1-weighted images show two sites of cerebellar polymicrogyria with cleft on the right (white arrows). (C4) sagittal T1-weighted image corresponding to the section on axial T2-weighted (C2) and T1-weighted (C3) images with a deep fissure and polymicrogyric cerebellar cortex (white arrow).

Midbrain, pons, medulla, cerebellar vermis and hemispheres were visually assessed for size and morphology. Diminished size of the aforementioned structures reflected hypoplasia (Fig. 1). The grade of involvement was further delineated as mild, severe or, in case of vermis–inferior vermian hypoplasia (Table 1). Dysplasia was considered if the midbrain–hindbrain structure was distorted and disorganized, if abnormal cerebellar (hemispheric or vermian) foliation and fissures were present (Fig. 2). For statistical analysis, MHM were divided into hypoplasia and dysplasia. MHM was attributed to either category (hypoplasia or dysplasia) if at least one of the posterior fossa structures (midbrain, pons, medulla, cerebellar vermis and hemispheres) was either hypoplastic or dysplastic. In cases, when hypoplasia and dysplasia of different posterior fossa structures coexisted, MHM was categorized as dysplastic.

**Table 1:** The patients with MHM, their spectrum and relationship to different MCD.

N	Sex	Age^a^	MCD category	MCD type	Midbrain	Medulla	Pons	Vermis	Cerebellar hemispheres
1	M	32	Proliferation	TS	N	N	N	IVH	N
2	M	23	Proliferation	TS	N	N	N	MH	N
3	W	11	Proliferation	TS	N	N	N	IVH	N
4	W	22	Proliferation	TS	N	N	N	MH	N
5	W	23	Proliferation	TS	N	N	N	N	D
6	M	16	Proliferation	MCRC	N	N	N	MH	MH
7	W	27	Proliferation	MCRC	N	N	N	SH	SH
8	W	8	Proliferation	HMGE	N	N	N	D	D
9	W	24	Proliferation	DT	N	N	N	MH	N
10	M	25	Proliferation	FCD II	N	N	N	MH	N

11	W	15	Migration	PNH	N	N	N	IVH	N
12	M	24	Migration	PNH	N	N	N	D	D
13	M	33	Migration	PNH	N	N	N	SH	MH
14	M	3	Migration	PNH	MH	N	N	D	D
15	W	29	Migration	PNH	N	N	N	MH	N
16	W	16	Migration	PNH	N	N	N	SH	SH
17	M	15	Migration	PNH	MH	N	N	D	D
18	W	39	Migration	PNH	N	N	N	IVH	N
19	M	39	Migration	PNH	SH	SH	SH	SH	SH
20	W	28	Migration	PNH	SH	SH	SH	SH	SH
21	W	44	Migration	SBH	N	N	N	IVH	N

22	M	11	Organization	PMG	N	N	N	D	D
23	W	11	Organization	PMG	N	N	N	D	D
24	W	30	Organization	PMG	MH	N	N	MH	N
25	M	35	Organization	PMG	MH	N	N	N	N
26	M	27	Organization	PMG	MH	N	N	N	N
27	M	45	Organization	PMG	MH	N	N	N	N
28	M	23	Organization	PMG	N	N	N	SH	MH
29	W	4	Organization	PMG	SH	N	N	N	N
30	W	34	Organization	PMG	MH	N	N	IVH	N
31	W	33	Organization	PMG	N	N	N	D	D
32	W	34	Organization	PMG	N	N	N	D	D
33	M	11	Organization	PMG	SH	MH	N	MH	N
34	W	6	Organization	PMG	N	N	N	MH	MH
35	M	20	Organization	PMG	SH	SH	N	N	N
36	M	34	Organization	PMG	SH	MH	N	MH	N
37	M	45	Organization	FCD I	N	N	N	MH	N
38	M	47	Organization	FCD I	N	N	N	D	D

*Abbreviations*: MHM: midbrain–hindbrain malformation; MCD: malformations of cortical development; W: woman; M: man; DT: developmental tumor; FCD II: focal cortical dysplasia type II; TS: tuberous sclerosis; MCRC: microcephaly; HMGE: hemimegalencephaly; PNH: periventricular nodular heterotopia; SBH: subcortical band heterotopia; PMG: polymicrogyria; FCD I: focal cortical dysplasia type I; N: normal; MH: mild hypoplasia; SH: severe hypoplasia; IVH: inferior vermian hypoplasia; D: dysplasia.

a Age at the time of this analysis.

MHM were classified into conventional categories (e.g. Rhombencephalosynapsis), whenever possible.

The cerebellum was regarded atrophic if it was diffusely small, shrunken with large fissures and if it underwent progressive volume loss over time (Patel and Barkovich, 2002).

Agreement between the two raters (GK, FK) was reached in 214/228 cases (Cohen's kappa coefficient, *κ* = 0.842, *p* < 0.0001).

Four out of 228 patients were excluded from the further analysis as they had progressive cerebellar atrophy (documented at least on two sequential MRIs), which was attributed to the longstanding use of phenytoin.

In 214 out of remaining 224 patients, 181 had normal midbrain–hindbrain structures, and 33 patients had MHM. In 10/224 disputed cases, a third evaluator, a neuroradiologist from another institution (SF), also blinded for clinical information, was consulted. The decision was made with two out of three votes: one out of ten patients was regarded as having normal midbrain–hindbrain structures, five out of ten – MHM, four out of ten patients were excluded from the study due to three different opinions.

Eventually, 220 patients were included in the study; 38/220 patients were considered having MHM; 182/220 – not having MHM.

Microcephaly was diagnosed in patients with head circumference remaining proportionately small with age and ranging between −4 and −12 standard deviation from the mean.

Hippocampal abnormalities were defined based on a categorization proposed by our group (Kuchukhidze et al., 2010):

Partially infolded/hypoplastic hippocampus: incompletely infolded or unfolded, vertically oriented, excessively thin hippocampus with distorted anatomy, missing internal structure, without signal change in T2 and FLAIR sequences.

Hippocampal sclerosis: atrophic hippocampus with missing internal structure, increased signal in T2 and FLAIR sequences.

Malrotated hippocampus: abnormally round, globular-shaped or pyramidal, vertically oriented rather than ovoid, incompletely rotated hippocampus without signal change in T2 and FLAIR sequences. Additional criteria were steep angle of collateral sulcus and particular triangular configuration of temporal horn (normal collateral sulcus angle is flat at the level of tale and body of hippocampus).

Enlarged hippocampus: hyperplastic hippocampus with distorted anatomy, missing internal structure and increased signal in T2 and FLAIR sequences.

Corpus callosum was assessed visually. Dysgenesis of corpus callosum was considered if it was either entirely or partially absent.

### Patients

Data of 220 patients (116 women/104 men, median age 28 years, interquartile range 20–44 years at the time of assessment) were retrospectively analyzed. Diagnosis of MCD was based on MRI in all patients. Patients with intelligence quotient (IQ) lower than 70 were classified as learning disabled. Seizure types and epilepsy syndromes were classified according to nomenclature of the ILAE Commission on Classification and Terminology, 1981, ILAE Commission on Classification and Terminology, 1989.

### Statistics

Categorical data were analyzed by means of 2 × 2 Chi-square test with Yates correction. Either Freeman-Halton extension of Fisher's exact probability test or Chi-square test with Yates correction were used for tables larger than 2 × 2. In case of significant differences, paired-wise comparisons were carried out by means of 2 × 2 Fisher's exact probability test or 2 × 2 Chi-square test with Yates correction. Non-categorical data (e.g., age at seizure onset) were first analyzed by Kruskal–Wallis test. Two-by-two comparisons were performed by means of Mann–Whitney test. Inter-rater agreement for assignment of MHM to different categories was assessed using Cohen's kappa coefficient (*κ*). Significance was set at *α* = 0.05. There were no missing data in the entire analysis.

In a preliminary data analysis, a series of univariate ANCOVA were performed to investigate whether age at the time of assessment, age at seizure onset and response to antiepileptic drugs were confounding factors with regard to clinical features (learning disability, neurological deficits, delayed developmental milestones etc.) when groups wMHM and w/oMHM were compared.

Multinomial logistic regression test was performed to determine whether the presence of bilateral MCD and dysgenesis of corpus callosum could be confounding factors for clinical presentation (epilepsy syndrome, learning disability, neurological deficit, developmental milestones, etc.) when groups wMHM and w/oMHM were compared.

### Ethical considerations

This is a retrospective non-invasive study, which does not require ethics committee approval according to the Austrian Law on Research. All patients gave informed consent for an MRI which was performed in the framework of diagnostic work up.

## Results

### MCD spectrum in the whole cohort (*n* = 220)

The most common MCD were polymicrogyria (21%), developmental tumors (15%), periventricular nodular heterotopias (14%), focal cortical dysplasia type II (14%), focal cortical dysplasia type I (13%) and tuberous sclerosis (TS) (10%). MCD were bilateral in 64% and unilateral – in 36% of patients. MCD was confined to temporal lobe in 40% of patients. Hippocampal abnormalities were observed in 31% and dysgenesis of corpus callosum – in 8% of patients with MCD. The clinical spectrum of the patients was as follows: 86% had focal epilepsy (most common were temporal lobe – 46% and multifocal – 24% epilepsies). Pharmacoresistant seizures were observed in 63% of patients; 18% – underwent epilepsy surgery. Neurological deficits were seen in 50%; learning disability – in 44% and delayed developmental milestones – in 40% of patients.

### Midbrain–hindbrain malformations (*n* = 38)

MHM were identified in 17% (38/220) of patients with MCD and epilepsy. Cerebellum (either vermis or hemispheres, or both) was affected in 84% of patients with MHM. Mild vermian hypoplasia was observed in 29%, severe vermian hypoplasia – in 16%, inferior vermian hypoplasia – in 16%, and vermian dysplasia – in 24% (Table 1). Cerebellar hemispheres were either dysplastic (26%) or hypoplastic (mild – 10%, severe – 10%) (Table 1). Merely hypoplasia was found in midbrain (mild – 18%, severe – 16%), medulla (mild–5%, severe – 8%) and pons (severe – 5%) (Table 1).

Patients with MHM-hypoplasia and MHM-dysplasia did not differ with regard to clinical and electrophysiological characteristics. MHM-hypoplasia and MHM-dysplasia did not vary with respect to the association with different MCD types, dysgenesis of corpus callosum and hippocampal abnormalities. Among those MHM attributed to the conventional categories were cerebellar polymicrogyria with clefts (*n* = 6) and rhombencephalosynapsis (*n* = 4).

### Comparison of wMHM and w/oMHM groups

The data presented in this section are detailed in Table 2.

**Table 2:** Comparison of wMHM and w/oMHM groups.

Demographical and clinical data	wMHM (*n* = 38)	w/oMHM (*n* = 182)	Test	*p*
Age in years, median (IQR)	25 (15–34)	30 (20–45)	M–W	**0.021**
Age at seizure onset in years, median (IQR)	5 (1–13)	12 (2–17)	M–W	**0.043**
Sex, W/M	19/19	97/85	Chi-square	0.841
Epilepsy syndrome, TLE/extra-TLE	11/27	91/91	Chi-square	**0.029**
Seizure outcome, seizure free/not seizure free	13/25	69/113	Chi-square	0.806
Status epilepticus, yes/no	2/36	13/169	Chi-square	1.0
Focal slowing on EEG, yes/no	27/11	137/45	Chi-square	0.740
Epileptiform discharges, yes/no	23/15	104/78	Chi-square	0.841
AED, monotherapy/polytherapy	18/20	90/92	Chi-square	1.0
Neurological deficit, yes/no	25/13	85/97	Chi-square	**0.049**
Delayed milestones, yes/no	26/12	63/119	Chi-square	**<0.001**
Learning disability, yes/no	27/11	70/112	Chi-square	**<0.001**
Hippocampal abnormalities, yes/no	20/18	49/133	Chi-square	**0.004**
Dysgenesis of corpus callosum, yes/no	10/28	7/175	Chi-square	**<0.001**
MCD category, I/II/III	10/11/17	99/25/58	Chi-square	**0.004**
MCD Laterality, unilateral/bilateral	14/24	116/66	Chi-square	**0.004**
MCD Location, temporal/extra-temporal	8/30	81/101	Chi-square	**0.012**
Tuberous sclerosis	5/33	18/164	Chi-square	0.548
Microcephaly	2/36	10/172	Chi-square	1.0
Hemimegalencephaly	1/37	9/173	Chi-square	0.532
Developmental tumors	1/37	32/150	Chi-square	**0.019**
Focal cortical dysplasia type II	1/37	30/152	Chi-square	**0.026**
Periventricular nodular heterotopia	10/28	20/162	Chi-square	**0.012**
Subcortical band heterotopia	1/37	3/179	Chi-square	0.680
Lissencephaly	0/38	2/180	N/A	N/A
Polymicrogyria	15/23	32/150	Chi-square	**0.003**
Focal cortical dysplasia type I	2/36	26/156	Chi-square	0.129

*Abbreviations*: wMHM: with midbrain–hindbrain malformation; w/oMHM: without midbrain–hindbrain malformation; IQR: interquartile range; W: women; M: men; TLE: temporal lobe epilepsy; AED: antiepileptic drugs; M–W: Mann–Whitney-test.

Chi-square test (two-tailed) was performed for either 2 × 3 or 2 × 2 tables.

Significant contrasts are marked in bold.

The rate of patients wMHM and w/oMHM differed significantly (*p* = 0.004) in three categories of MCD (category I – due to abnormal neuronal proliferation; category II – due to abnormal neuronal migration; and category III – due to abnormal neuronal late migration/organization). Patients with MCD of category II (31%) were more commonly associated with MHM compared to those with MCD of category I (9%, *p* = 0.004). Likewise, there was a strong link between MHM and MCD of category III (23%) compared to MCD of category I (9%, *p* = 0.020). There was no significant difference with respect to the rate of MHM when categories II (31%) and III (23%) of MCD were compared. Patients wMHM compared to those w/oMHM had more commonly polymicrogyria (39% vs. 17.5%, *p* = 0.003) and periventricular nodular heterotopias (26% vs. 11%, *p* = 0.012). Patients wMHM versus those w/oMHM were less frequently associated with developmental tumors (3% vs. 17%, *p* = 0.019) and focal cortical dysplasia type II (3% vs. 16%, *p* = 0.026).

Patients wMHM had more often extensive bilateral MCD (e.g. bilateral perisylvian polymicrogyria, bilateral periventricular nodular heterotopia located along lateral ventricles) – 63% in comparison to those w/oMHM – 36% (*p* = 0.004).

Extra-temporal location of MCD was more common in patients wMHM – 79% compared to those w/oMHM – 55% (*p* = 0.012). Similarly, extra-temporal lobe epilepsies were more frequent in wMHM patients – 71% vs. those w/oMHM – 50% (*p* = 0.029).

There were higher rates of callosal dysgenesis (26% vs. 4%; *p* < 0.001) and hippocampal abnormalities (52% vs. 27%; *p* < 0.001) in wMHM group compared to w/oMHM group. Patients wMHM compared to those w/oMHM had stronger association with bilateral hippocampal abnormalities (39% vs. 13%; *p* < 0.001) and hypoplastic/partially infolded hippocampus (31%, vs. 12%; *p* = 0.005).

Patients wMHM were younger compared to those w/oMHM (median 25 years vs. 30 years; *p* = 0.010) at the time of this analysis. Patients wMHM compared to those w/oMHM had an earlier age at seizure onset (median 5 years vs. 12 years; *p* = 0.043).

Patients wMHM compared to those w/oMHM had higher rates of learning disability (71% vs. 38%; *p* < 0.001), delayed developmental milestones (68% vs. 35%; *p* < 0.001) and neurological deficits (66% vs. 47%; *p* = 0.049).

Results of a preliminary data analysis (series of univariate ANCOVA and multinomial logistic regression test) indicated that age at the time of assessment, age at seizure onset, response to antiepileptic drugs, presence of bilateral MCD as well as dysgenesis of corpus callosum were not significant confounding factors with regard to the rate of learning disability, neurological deficits and delayed developmental milestones when groups wMHM and w/oMHM were compared.

The groups (wMHM and w/oMHM) did not differ in their response to antiepileptic treatment, seizure outcome, seizure types, EEG abnormalities and rate of status epilepticus.

## Discussion

In this study, we aimed to investigate the spectrum of MHM in patients with MCD and epilepsy, to explore associations between MHM and MCD and to compare clinical and imaging features in two groups of patients: (i) with MCD and MHM and (ii) with MCD and without MHM.

We report 17% of MHM in a large series of 220 patients with MCD and epilepsy. We found more severe morphological (extensive bilateral MCD, hippocampal abnormalities, callosal dysgenesis) and clinical (learning disability, neurological deficits, developmental delay and earlier age at seizure onset) phenotypes in patients with MHM and MCD compared to those who had MCD without MHM.

As hypothesized, significant differences were found in association between MHM and various categories of MCD. MHM were more commonly linked to the later developmental disorders – MCD due to abnormal neuronal migration and abnormal neuronal organization compared to the earlier MCD, due to abnormal neuronal proliferation. This association could be considered an indication of the common pathophysiological background of late cerebral migrational–organizational disorders and MHM. Development of cerebellum starts at about four weeks of gestation and extends to almost 20 months of postnatal age (Limperopoulos and du Plessis, 2006, ten Donkelaar et al., 2003). The migration of cerebellar and cerebral cortical neurons starts simultaneously at about 8th week of gestation and their organization and lamination continues into postnatal age (Gleeson and Walsh, 2000, Limperopoulos and du Plessis, 2006, ten Donkelaar et al., 2003). The injury to highly vulnerable migrating cells from insults such as viruses, toxins, hemorrhages may disturb subsequent cerebellar or cortical development. Another mechanism leading to both, supratentorial cortical malformations and MHM, may be genetic. Indeed, a recent study on midbrain–hindbrain involvement in lissencephalies demonstrated clear associations of specific MHM phenotypes with lissencephaly subtypes. The association of lissencephaly pattern with the extent and severity of midbrain–hindbrain involvement assisted in predicting the mutations responsible for the developmental disorder (Barkovich et al., 2009, Jissendi-Tchofo et al., 2009).

In our study, we could not validate phenotype-genotype correlations since only very few of our patients underwent genetic testing. However, our findings may guide further genetic research.

MHM-hypoplasia was the most common in our series falling in line with another cross-sectional retrospective study in which Dandy-Walker malformation, marked by cerebellar hypoplasia, comprised 27% (19/70) of patients with cerebellar malformations (Patel and Barkovich, 2002).

Cerebellum plays an important role in cognition. Patients with cerebellar disorders, either congenital or acquired, may have cognitive impairment (Timmann and Daum, 2010). Cerebellar malformations could be associated with affective and social disorders as well as neuropsychological deficits involving mainly executive functions, visuospatial and linguistic abilities (Tavano et al., 2007). Motor deficits are usually less severe than cognitive impairment in the patients with cerebellar developmental malformations (Tavano et al., 2007). According to our findings learning disability and developmental delay were more frequently observed in patients wMHM compared to those w/oMHM. Patients in the wMHM group had a high frequency of bilateral MCD, which could potentially cause abnormal cognitive status (Berg et al., 2009). However, in our data analysis, bilateral MCD was not a significant confounding factor when rates of learning disability and developmental delay were compared in wMHM and w/oMHM groups. Higher rate of learning disability in our patients wMHM compared to those w/oMHM could not be attributed to epilepsy alone, as there was no difference between the groups in terms of duration of epilepsy and the response to antiepileptic drugs.

Dysgenesis of corpus callosum, which occurred frequently in our wMHM group, is a very heterogeneous condition. It could be isolated or occur as a feature of numerous congenital syndromes associated with MCD as well as MHM (Barkovich and Norman, 1988, Tang et al., 2009). Disorders of corpus callosum present clinically with cognitive impairment, developmental delay and seizures (Moes et al., 2009). In patients with callosal dysgenesis, hippocampi are frequently hypoplastic as in our series (Barkovich and Norman, 1988). Indeed, hypoplastic/partially infolded hippocampus (15%, 34/220), the commonest hippocampal abnormality in the whole series, which occurred bilaterally in the majority of cases (91%, 31/34), was more commonly seen in wMHM group compared to w/oMHM group. MR images of hypoplastic/partially infolded hippocampus resembled MR appearance of hippocampus at early gestational stages, suggesting its developmental abnormality (Kier et al., 1997).

The main limitation of this study is related to the selection bias – we studied MHM in a group of patients with MCD and epilepsy. Therefore, we cannot draw conclusion on the whole spectrum of MHM.

Another shortcoming of this study is the fact that MCD diagnosis was largely based on MRI and histological confirmation was available only a relatively small proportion (40/220; 18%) of patients who underwent epilepsy surgery. In general, MCD could be reliably diagnosed on MRI (Kuzniecky and Jackson, 2005); the most notorious for being missed on conventional MRI is FCD type I comprising about 25% of cases in imaging-histology correlative studies on large series of patients (Krsek et al., 2008). In all cases of FCD type I included in our series (*n* = 28), a histological diagnosis of postsurgical specimens was available.

The findings of this study suggest that the presence of MHM in patients with MCD and epilepsy could be an indicator of a severe morphological and clinical phenotype. Our results may have clinical implication with regard to the sub-syndrome classification and may contribute to subsequent genetic and basic research in the field.
